# Mobile application-based oximetry: a potential toolfor appropriate referral of patients with respiratory symptoms examined via telemedicine

**DOI:** 10.31744/einstein_journal/2023AO0025

**Published:** 2022-12-15

**Authors:** Fernanda Vieira Paladino, Tarso Augusto Duenhas Accorsi, Bárbara Yasmin Gueuvoghlanian-Silva, Marcia Aparecida de Almeida, João Carlos Barbosa, Miguel Almeida de Oliveira, Carlos Henrique Sartorato Pedrotti, Karine De Amicis, Claudio Luiz Lottenberg, Eduardo Cordioli

**Affiliations:** 1 Hospital Israelita Albert Einstein São Paulo SP Brazil Hospital Israelita Albert Einstein, São Paulo, SP, Brazil.

**Keywords:** Oximetry, Dyspnea, Signs and symptoms, Respiratory tract diseases, Telemedicine, Referral and consultation, Artificial intelligence

## Abstract

**Objective:**

To calculate the positive likelihood ratio to determine whether telemedicine is able to optimize referral to the emergency department.

**Methods:**

Unicenter study with 182 consecutive patients admitted to *Hospital Israelita Albert Einstein* due to respiratory symptoms. All patients were submitted to oxygen saturation measurement using the standard method Welch Allyn finger device vital sign monitor and a 2-minute evaluation (Binah.ai mobile application). The reproducibility of oxygen saturation measurements made with both methods was investigated using interclass correlation coefficients and analysis of dispersion. Bland-Altman plots were constructed and kappa concordance coefficients used to examine data normality. Accuracy was also estimated.

**Results:**

Oxygen saturation measurement differences between methods were ≤2% in more than 85% of cases. The mean difference (bias) between methods was near zero (0.835; Bland-Altman analysis). Oxygen saturation measurements made using the Binah.ai mobile application had an average ability to detect patients with altered oxygen saturation levels compared to the conventional method (ROC analysis). The positive likelihood ratio of the mobile application was 6.23.

**Conclusion:**

Mobile applications for oxygen saturation measurement are accessible user-friendly tools with moderate impact on clinical telemedicine evaluation of patients with respiratory symptoms, and may optimize referral to the emergency department.

## INTRODUCTION

Respiratory symptoms are common, have multiple causes and may be associated with a wide range of potentially life-threatening differential diagnoses. Patients with respiratory symptoms account for a large proportion of the emergency department (ED) caseload.^(1)^ According to the World Health Organization (WHO), respiratory diseases cause 59 million deaths annually and are the third leading cause of death among adults and children.^(2)^ About one-third of outpatient and emergency care cases and hospital admissions involve patients with respiratory diseases.^(3)^ The large number of patients seeking the health system due to acute respiratory complaints emphasizes the need of accurate, cost-effective and early out-of-hospital assessment. The primary objective of initial patient evaluation is to identify red flags for proper referral for emergency care. The major signs of high-risk respiratory conditions are dyspnea, high respiratory rates and low oxygen levels on oximetry.^(4)^

During the COVID-19 pandemic, telemedicine (TM) has become a critical resource due to economic capacity to serve large populations and enable immediate health actions safely.^(5)^ Government-imposed quarantine has turned TM into a virtual ED, in order to promote appropriate referral for face-to-face emergency care, minimize viral spread, prevent further contamination and avoid overcrowding and health system overload.^(6,7)^ Clinical assessment of several conditions via telemedicine has proven highly accurate and is associated with less referrals to the ED overall.^(8)^ However, remote assessment of patients with dyspnea is limited, particularly due to constraints related to vital sign extraction, especially oximetry data. In a recent study with 500 patients with acute respiratory symptoms assessed via TM during the COVID-19 pandemic, only 16 (3.2%) patients had a pulse oximeter at home.^(9)^

A feasible RGB camera-based method (photoplethysmography, PPG) for non-contact vital sign measurement has recently been reported.^(10-12)^ This method combines color change signals sampled from different regions of the face using estimated magnitudes of red cell aggregation dictated by blood perfusion with reflected light intensity in each of these regions. Variations in averaged color signals are then compared to improve camera-based signal-to-noise ratio (SNR) estimates. A low-cost, user-friendly mobile application solution has been developed (Binah.ai) to measure oxygen levels using a frontal smartphone camera.

## OBJECTIVE

To calculate the positive likelihood ratio to determine whether telemedicine is able to optimize referral to the emergency department.

## METHODS

### Technology

Binah.ai is a mobile application solution that uses a combination of signal processing and AI technologies with a proprietary mathematical backend server to analyze the skin of the upper cheek region of the face through the front camera of smartphones.

Binah.ai application uses signal processing AI technologies to capture video images and analyzes data collected using PPG techniques. This methodology provides real-time measurements of end-user physiological parameters, including oxygen saturation levels (SpO_2_), heart rate (HR), pulse (PR) and respiratory (RR) rates. Binah.ai application employs a novel technology relative to other existing applications. In this application, ambient light RGB signals extracted from face videos in real-time are used, with no need of cloud servers or internet connection.

### Procedures

This study was carried out at *Hospital Israelita Albert Einstein* (HIAE) and was approved by the Institution’s Research Ethics Committee (CAAE: 32352320.9.0000.0071; # 4.065.552). All participants gave written informed consent and study risks were fully explained. Patients aged over 18 years with normal (≥95%) or low (≤94%) SpO_2_ were recruited from the ED, intensive care unit (ICU) and hospitalization ward between September and November 2020. Intubated and unconscious patients were excluded.

This study validated a new technology for SpO_2_ monitoring, which was benchmarked against the conventional method (Welch Allyn monitor). The application was downloaded to three smartphones (one in each recruitment area) for SpO_2_ measurement. Patient were positioned in front of the mobile phone and the application automatically started to measure oxygen saturation. The analysis took 1 to 2 minutes to complete. Measurements were simultaneously made using a Welch Allyn monitor. Data were collected by the health professional in charge of patient care.

To ensure that no information would be stored in the Binah.ai cloud, the application was activated only when the cell phone was offline. Hence, the company did not have access to data collected during tests. The application was also deleted after use to prevent data from being upload into the system.

### Statistical analysis

Numerical variables were expressed as minimum and maximum values, means, standard deviations (SD), medians and quartiles. Categorical variables were expressed as absolute and relative frequencies.

The reproducibility of SpO_2_ measurements made using both methods was investigated using intra-class correlation coefficients (ICC) and analysis of dispersion. Bland-Altman plots were constructed, and kappa concordance coefficients used to examine data normality. Sensitivity, specificity, positive (PPV) and negative (NPV) predictive values, accuracy and likelihood ratios were calculated to determine the ability of the new application to accurately measure SpO_2_. The 95% confidence interval (95%CI) was considered in data analysis.

The ability of SpO_2_ measurements made with the new application to detect changes in oxygen saturation was investigated using ROC curves (Receiver Operating Characteristic). Statistical analyses were performed using SPSS and R packages, with a level of significance of 5%.

## RESULTS

The sample comprised 182 patients. There were no refusals to participate, data loss or technical problems. Oxygen saturation (%) was measured using the conventional method and the Binah.ai mobile application (Table 1).

**Table 1:** Oxygen saturation measurements made using a vital sign monitor and the Binah.ai mobile application

Oxygen saturation	Conventional method (n=182)	Binah.ai mobile application (n=182)	Difference between methods
Mean (SD)	97.0 (2.7)	97.8 (2.5)	0.8 (2.1)
Median (Q1; Q3)	97.5 (96.0; 99.0)	99.0 (96.0; 100.0)	1.0 (0.0; 2.0)
Minimum-maximum	83.0-100.0	85.0-100.0	-8.0-12.0

SD: standard deviation; Q1: first quartile; Q3: third quartile.

Differences in SpO_2_ measurements ranged from -8 to 12%. Fifty-two (28.6%) SpO_2_ values were identical, 27 (14.8%) were higher with the Binah.ai mobile application and 103 (56.6%) were higher with the conventional method (Table 2).

**Table 2:** Differences in oxygen saturation measurements between the conventional method and the Binah.ai mobile application

Difference between methods	Oxygen saturation n (%)
>-3	7 (3.8)
-2	3 (1.6)
-1	17 (9.3)
0	52 (28.6)
1	56 (30.8)
2	27 (14.8)
>3	20 (11.0)

Intra-class correlation coefficients were calculated to determine the reproducibility of SpO_2_ measurements made with both methods. Findings revealed moderate reproducibility of oxygen saturation measurements (ICC: 0.626, 95%CI: 0.484-0.727; p<0.001).

**Figure 1:**
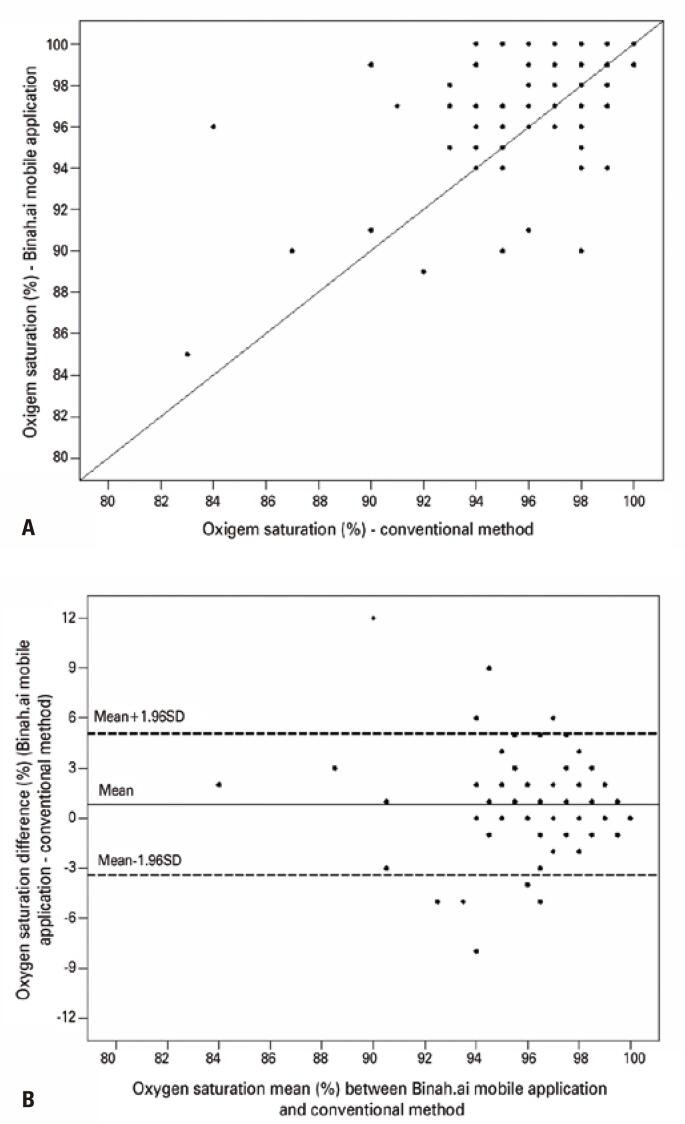
Oxygen saturation measurement reproducibility assessment: (A) Correlations between oxygen saturation measurements made using the Binah.ai mobile application and the conventional method; (B) Bland-Altman plots of oxygen saturation measures made using the Binah.ai- mobile application and the conventional method

Dispersion graphs and Bland-Altman plots were constructed to examine the reproducibility of SpO_2_ measurements obtained using each method (Figure 1). This analysis revealed a significant positive correlation between SpO_2_ values measured with the Binah.ai mobile application and the conventional method (r=0.66, p<0.001). The mean difference (bias) between SpO_2_ measurement methods was 0.835%, with 95% limits of agreement (mean±1.96 SD) ranging from -3.409% to 5.079%.

Oximetry readings were classified as normal according to normality standards (SpO_2_ up to 95%). Kappa coefficients were calculated to examine the agreement between SpO_2_ measurement methods (Table 3). This analysis revealed reasonable agreement between methods (Kappa: 0.278, 95%CI: 0.066-0.490; p=0.002).

**Table 3:** Classification of oxygen saturation measurements obtained using the Binah.ai-mobile application and the conventional method

Binah.ai mobile application	Conventional method		Total (%)

	Altered (%)	Normal (%)	
Oxygen saturation			
Altered	6 (3.3)	7 (3.8)	13 (7.1)
Normal	16 (8.8)	153 (84.1)	169 (92.9)
Total	22 (12.1)	160 (87.9)	182 (100.0)
Agreement	159 (87.4)		
Kappa coefficient (95%CI)	0.278 (0.066-0.490)		

95%CI: 95% confidence interval.

Sensitivity, specificity, PPV, NPV, positive likelihood ratios (+LR), negative likelihood ratios (-LR) and accuracy were calculated to compare the classification of SpO_2_ measurements obtained using the Binah.ai mobile application and the conventional method (Table 4).

**Table 4:** Performance measures of the Binah.ai mobile application and the conventional method regarding oxygen saturation classification

Performance measures	Oxygen saturation (n=182) 95%CI
Sensitivity	0.273 (0.087-0.459)
Specificity	0.956 (0.925-0.988)
PPV	0.462 (0.191-0.733)
NPV	0.905 (0.861-0.949)
+LR	6.23 (2.30-16.86)
-LR	0.76 (0.59-0.98)
Accuracy	0.874 (0.825-0.922)

PPV: positive predictive value; NPV: negative predictive value; +LR: positive likelihood ratio; -LR: negative likelihood ratio; 95%CI: 95% confidence interval.

Oxygen saturation measurements made using the Binah.ai mobile application had low sensitivity (27.3%) and high specificity (95.6%). Comparison against values measured using the conventional method (gold-standard) revealed low PPV (46.2%) and high NPV (90.5%). The +LR and -LR were 6.23 and 0.76 respectively, and the accuracy was high (87.4%).

**Figure 2:**
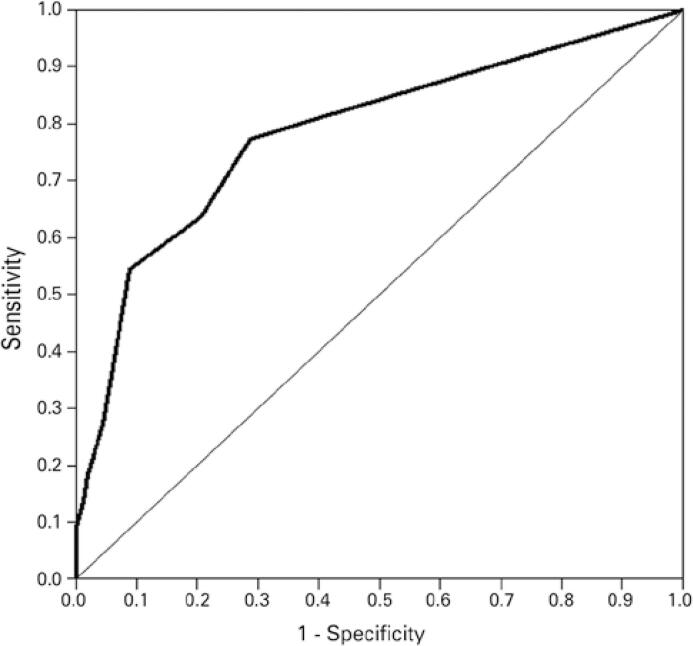
ROC curve of oxygen saturation measurements obtained using the Binah.ai mobile application and classification of oxygen saturation values according to the conventional method (n=182)

A ROC curve was constructed to investigate the performance of measurements obtained using the Binah.ai mobile application to detect patients with changes in SpO_2_ according to measurements made with the conventional method. The observed value of the area under the curve was 0.86 (95%CI: 0.672-0.900) (Figure 2).

Area under the curve: 0.86 (95%CI: 0.672-0.900).

## DISCUSSION

In patients with respiratory symptoms, the primary aim of clinical assessment is to detect dyspnea and warning signs suggestive of a poor prognosis to determine the need of immediate ED referral. More and more, such patients tend to be evaluated in out-of-hospital settings using TM.

Dyspnea is often interpreted as a red flag and need of face-to-face medical consultation. In a study with 166,908 prehospital patients referred to the ED, 11.9% of referrals were due to dyspnea. Half of those patients were admitted, one-third were treated in ICU and 15% required invasive mechanical ventilation. The in-hospital mortality rate was 10%. Almost half of acute dyspnea cases seen in the ED were due to heart failure (HF), pneumonia or chronic obstructive pulmonary disease (COPD).^(13)^

Delivery of care to patients with respiratory symptoms is not simple. There is a need to maintain face-to-face services in crowded EDs, which has become a significant problem during the COVID-19 pandemic.^(14)^ The pandemic has brought an urgent need to adopt TM practices. Such practices have proven fundamental in times of social isolation to reduce infection and ensure appropriate care and monitoring of patients with pre-existing diseases and morbidities such as diabetes, hypertension and cardiovascular or chronic respiratory diseases, who chose not to visit hospitals.^(15)^

Studies investigating causes of emergency service overcrowding have emphasized the high volume of nonspecific presentations and limited access to primary care.^(16)^ Overcrowding of emergency services ultimately leads to a poorer prognosis in emergency cases.^(17)^Early medical assessment of patients with acute symptoms, particularly in prehospital settings, is an interesting strategy to reduce ED overcrowding.^(18)^ In this scenario, TM stands out as a promising strategy, which has gained momentum during the COVID-19 pandemic.

Telemedicine allows patients to connect with healthcare providers remotely and plays an essential role in prehospital assessment, leading to more accurate ED referrals and avoiding face-to-face consultations in low-risk situations.^(19)^ There two critical constraints associated with TM assessment of patients with dyspnea: accuracy of remote physical examination findings and diagnostic flow with ancillary tests.

Despite potential limitations of remote assessment, there is enough evidence to support the similarity of clinical impression between face-to-face and remote consultations. A systematic review of 33 prospective cohort studies with 13,833 pediatric patients with suspected community-acquired pneumonia (CAP) seeking emergency care, no physical examination signs with high positive or negative likelihood ratio were detected. In that review, signs more strongly associated with CAP were elevated respiratory rates and hypoxemia (defined as SpO_2_ lower than or equal to 96%), with +LR of 2.1 and 2.8 respectively.^(20)^ As to adult patients with suspected COVID-19, a meta-analysis of 16 studies including 7,706 participants experiencing acute symptoms during the pandemic failed to identify signs that could accurately confirm or rule out the disease.^(21)^

Medical reasoning combining clinical and complementary information in the diagnostic flow is more important than whether medical assessment is carried out in person or remotely.^(22)^ Appropriate diagnostic flow starts with pre-test hypotheses based on clinical impression and formulated according to medical knowledge, disease prevalence in the population and data from studies. Even with sufficient clinical data, the accuracy of clinical diagnostic is relatively low, and must be supported by other pieces of information, particularly ancillary test results.^(23)^ Combination of clinical features and test results in decision trees improves diagnostic accuracy. In emergency care settings, patients with dyspnea are immediately submitted to clinical evaluation complemented by oximetry. This approach is associated with enhanced identification of life-threatening circumstances, but not with actual diagnosis.^(24)^ Multiple tests are performed in sequence until a high post-test probability of diagnosis is attained.^(25)^ Also, confirmation of hypoxemia implies early oxygen therapy and improved prognosis.^(26)^

Massive accumulation of evidence indicates similar performance of TM and face-to-face assessment to collect clinical data from patients with dyspnea for estimation of pre-test probabilities. For example, in prehospital settings, the only physical examination parameter associated with admission was elevated respiratory rate, which can be easily measured remotely.^(13)^ However, a significant limitation of remote out-of-hospital assessment is the impact on pre-test probabilities with new information from ancillary tests. Despite of the large number of ancillary tests available, the vast majority of bedside tests have limited diagnostic value when used in isolation and are poorly correlated with prognosis. Oxygen saturation is the single most relevant piece of information that adds value to clinical assessment of respiratory rate, especially as an indicator of the need to perform oximetry in the ED. This is the rationale of this study.

Binah.ai startup developed a novel technology, a mobile application aimed at measuring oxygen saturation via a 2-minute real-time assessment of the patient’s face using the front camera of smartphones. Smartphones are widely available these days, even in low-income populations. Hence, new software applications can be applied in health care. In this study, the Binah.ai application was benchmarked against the conventional in-person monitoring method (Welch Allyn monitor). In more than 85% of measurements, oxygen saturation differences between methods were ≤2%. This is certainly a good result. However, this finding must be addressed from a clinical standpoint, since these even small differences may have significant impacts on patients with low blood oxygen levels.

The wide variation between methods (-8 to 12%) is also important. Although a large proportion of SpO_2_ measurements were identical to (28.6%) or lower (56.6%) than the gold-standard, 14.8% of Binah.ai readings were higher, which may lead to unnecessary patient referral for emergency care.

Bland-Altman analysis revealed a relatively high 95% limit of agreement, with a mean difference (bias) between oxygen saturation measurement methods near zero (0.835). Use of more data points may drive this limit down. According to findings of ROC analysis, oxygen saturation measurements made using the Binah.ai mobile application had an average performance to detect patients with altered SpO_2_ compared to the conventional method. In a validation study of vital sign measurements made with two devices, smartwatch technology was not accurate enough compared to a portable health device and failed to meet predefined criteria for oxygen saturation.^(27)^ Advantages of the Binah.ai mobile application highlighted in this study include lack of need of other devices, such as smartwatches or portable oxygen saturation measurement devices, immediate reading, and easy download during TM consultations.

With respect to accuracy, results obtained are similar to several commonly used ancillary bedside tests: low sensitivity, high specificity and moderate +LR. The +LR in this study (6.23) increased pre-test probability by more than 30%.^(28)^ The most popular bedside diagnostic tests used to assess dyspnea have low to moderate positive likelihood ratio for the most prevalent diagnostic hypotheses. For example, the +LR of chest X-ray for detection of heart failure (HF) is 4.8. The -LR of most bedside tests is also not low enough for diagnostic exclusion (*e.g.*, brain natriuretic peptide of 0.11 for HF).^(29)^ Another study evaluating the Samsung application reported high correlations with arterial blood gas measurements (0.97; 95%CI: 0.95-0.98). However, performance in patients with hypoxia was not as good.^(30)^

In the specific context of patients with respiratory symptoms assessed via TM, low oxygen saturation measured remotely using Binah.ai mobile application in patients with elevated respiratory rates implies very high post-test probability of a life-threatening situation and indisputable need of ED referral. In the case of patients with questionable symptoms (*e.g.*, 50% pre-test probability of hypoxemia) and absence of red flags in remote assessment, oxygen desaturation revealed by the application significantly increases post-test probability and may lead to changes in medical approach in several cases. Weak -LR shows that application-based oximetry should not be used outside the clinical context.

As strengths of this study, the Binah.ai application provides users with a tool to measure oxygen saturation using only a smartphone, with no need to buy other devices, which can make the solution available anywhere. The application does not require an internet connection to work, provided it has been downloaded to a smart phone. However, this study has some limitations. Firstly, it was not possible to obtain a large enough sample for comparison of numerical values. Hence, values could not be categorized as normal or abnormal. Still, this study was able to demonstrate that categorical data can be used to inform decisions based on TM assessment. Secondly, patient assessment was not limited to TM and clinical follow-up, and no comparisons were made with similar technologies. As to what customers who detected low oxygen saturation should do, ethical, legal and liability issues can be discussed. However, this matter applies to all home devices. Despite the limitations, the Binah.ai application is thought to offer more benefits than harms to users.

## CONCLUSION

Mobile applications for oximetry measurement are accessible user-friendly tools which can be used to support clinical telemedicine evaluation of patients with respiratory symptoms and potentially enhance appropriate referral to the emergency department. However, these applications should not be used outside a clinical context. The application evaluated in this study was sufficiently correlated with the control device. This study provides evidence that the application can be used to measure oxygen saturation with high accuracy compared to a hospital bedside pulse oximeter, and could be a useful clinical tool for healthcare practitioners and telemedicine patients.
